# Cargo Release from Polymeric Vesicles under Shear

**DOI:** 10.3390/polym10030336

**Published:** 2018-03-19

**Authors:** Yingying Guo, Luca di Mare, Robert K. Y. Li, Janet S. S. Wong

**Affiliations:** 1 Department of Mechanical Engineering, Imperial College London, London SW 7 2AZ, UK; yingying.guo@imperial.ac.uk; 2 Department of Engineering Science, University of Oxford, Oxford Thermofluids Institute, Oxford OX2 0ES, UK; 3 Department of Materials Science and Engineering, City University of Hong Kong, Tat Chee Ave, Kowloon Tong, Hong Kong, China; aprkyl@cityu.edu.hk

**Keywords:** polymer vesicles, shear-induced, cargo release, confined shear, additive carriers, soft nanoadditives

## Abstract

In this paper we study the release of cargo from polymeric nano-carriers under shear. Vesicles formed by two star block polymers—$A_{12}B_{6}C_{2}$ (ABC) and $A_{12}B_{6}A_{2}$ (ABA)—and one linear block copolymer—$A_{14}B_{6}$ (AB), are investigated using dissipative particle dynamics (DPD) simulations. A- and C-blocks are solvophobic and B-block is solvophilic. The three polymers form vesicles of different structures. The vesicles are subjected to shear both in bulk and between solvophobic walls. In bulk shear, the mechanisms of cargo release are similar for all vesicles, with cargo travelling through vesicle membrane with no preferential release location. When sheared between walls, high cargo release rate is only observed with ABC vesicle after it touches the wall. For ABC vesicle, the critical condition for high cargo release rate is the formation of wall-polymersome interface after which the effect of shear rate in promoting cargo release is secondary. High release rate is achieved by the formation of solvophilic pathway allowing cargo to travel from the vesicle cavity to the vesicle exterior. The results in this paper show that well controlled target cargo release using polymersomes can be achieved with polymers of suitable design and can potentially be very useful for engineering applications. As an example, polymersomes can be used as carriers for surface active friction reducing additives which are only released at rubbing surfaces where the additives are needed most.

## 1. Introduction

Nano-carriers are nanoscale containers which can enclose small molecules and protect inclusions from reactions with the outer environment or help separate incompatible components in different compartments [1]. Vesicles are a popular type of nano-carriers with membranes made of lipids or polymers [2] which can encapsulate cargo such as drugs [3,4,5,6] or agrochemicals [7] during formation. These cargo could then be released steadily or quickly (controlled release) when desired conditions are reached. Cargo release can be triggered by a number of factors, such as temperature [5,8,9,10], pH value [3,11,12,13], light [14,15], shear stress [4,13,16,17,18,19], voltage [6], solid support [20] or magnetic field [21].

Mable et al. [8,9] encapsulated silica nanoparticles with vesicles made of poly(glycerol monomethacrylate)-poly(2-hydroxypropyl methacrylate) diblock copolymers. They showed that vesicles encapsulating 5% *w*/*w* silica nanoparticles underwent a vesicle-to-micelle transition and released their cargo upon cooling. Wu et al. [6] studied drug release from polymersomes (vesicles made of polymers) using electric fields. They found that the polymersomes partially collapsed and expelled their cargo after being electrochemically oxidized. Lomas et al. [22] studied polymersomes for controlled release of DNA under pH stimulus. By changing pH value from the physiological value to the cellular endocytic value, polymersomes dissolved into individual polymer chains and released the cargo. In the studies mentioned so far, the integrity of the membrane is permanently lost upon release of the cargo.

Changing vesicle membrane permeability can also be an effective way to control release whilst preserving the integrity of the carriers. Forming pores on the membrane is one way to tune membrane permeability. In Mable et al. [8], the membranes of vesicles with 20% *w*/*w* encapsulated silica nanoparticles perforated upon cooling, allowing the release of the cargo. Liu et al. [5] investigated temperature sensitive polymersomes made of poly(*N*-vinylcaprolactam)*_n_*-poly(dimethylsiloxane)_65_-poly(*N*-vinylcaprolactam)*_n_* (PVCL*_n_*-PDMS_65_-PVCL*_n_*) for controlled anticancer drug delivery. By increasing temperature to or above that of the PVCL phase transition, PVCL block chains gradually collapsed, leading to an increase in membrane porosity. Wang et al. [14] synthesized a polymersome with a membrane of switchable permeability using an amphiphilic poly(ethylene oxide)-b-PSPA (PEO-b-PSPA) diblock copolymers, where SPA is a spiropyran (SP)-based monomer and is light sensitive. By changing the wavelength of irradiation, the hydrophobic SP moieties transform to hydrophilic zwitterionic merocyanine (MC) moieties and change the permeability of the vesicle membrane. Cargo were released when hydrophilic MC moieties dominated.

Mechanical shear is also known to affect cargo release but the behaviour of polymersomes under shear has rarely been studied. Recently, Poschenrieder et al. [19] studied polymersome stability in stirred-tank reactors. They found that the dye-encapsulated polymersomes were stable under the conditions typically encountered in reactors for the biological industry and that dye release occurred mainly through membrane defects. More work has been done with liposomes and potentially the behaviour of liposomes and polymersomes may be similar. Some researchers suggested that the formation of transient pores allows cargo to be released under shear [4,16,17]. Other mechanisms are also possible. Bernard et al. [18] studied vesicles made of lipids (EPC) and detergent molecules (Brij76) under shear. When the vesicle was deformed, the detergent molecules, which have a larger spontaneous curvature, aggregated at the locations with the largest curvature on the membrane and induced pore formation. Researchers have also tried to emulate in polymersomes the transport mechanisms of living cells [23] by adding transmembrane proteins to enhance membrane permeability [23,24,25]. However, inserting channel proteins to fully span the whole polymersome membrane is challenging [26,27,28] because polymersomes possess thicker and more viscous [29] membranes than liposomes.

Polymersomes possess distinct advantages over liposome including better mechanical and structure stability [10,19,22,30,31], tunable architectural parameters [32,33] and properties [19,29,30], reduced permeability [10,26,31]. While research on vesicles have mainly focused on biological applications, the mechanical stability of polymeric vesicles open opportunities for their use in engineering applications, where targeted cargo delivery can be useful. Such applications include lubrication where local shear rate is high (up to 10^8^ s^−1^). One can envision additives encapsulated inside polymeric vesicles being transported to rubbing contacts and released where critical conditions are met. If successful, this may allow additives that are previously not suitable due to their poor solubility to be encapsulated in polymersomes, which will then be dispersed in base lubricants, leading to larger range of available lubricant additives. The amount of additives in lubricants can also be reduced, thereby reducing costs and potential for pollution. With this prospect in mind, this paper examines how cargo are released from polymersomes at high shear rates. We investigate how the cargo release mechanisms are affected by the architecture of the polymer chains, the structure of the polymersomes and the presence of solid surfaces. Three polymer architectures are used: a familiar AB diblock copolymer, an ABA terpolymer and finally an ABC terpolymer.

## 2. Methodology

Dissipative particle dynamics (DPD) simulation was used to study the cargo release process of polymeric vesicles under shear. DPD is a particle-based mesoscopic simulation technique first introduced by Hoogerbrugge and Koelman [34]. In DPD simulations all polymers are coarse-grained and are composed of DPD particles of equal size (see Table 1). The motion of each DPD particle is governed by Newton’s equation of motion. The total force acting on each particle i is ${\vec{f}}_{i\;}={\vec{F}}_{ij}^{C}+{\vec{F}}_{ij}^{D}+{\vec{F}}_{ij}^{R}+{\vec{F}}_{ij}^{S}$. The conservative force ${\vec{F}}_{ij}^{C}$, dissipative force ${\vec{F}}_{ij}^{D}$, random force ${\vec{F}}_{ij}^{R}$ [35,36] are pairwise contributions and become effective when the distance between two beads *i* and *j* is within the cut-off radius $r_{c}$. The conservative force ${\vec{F}}_{ij}^{C}$ is a soft-repulsive force and is given by

(1) $${\vec{F}}_{ij}^{C}=\{\begin{array}{c} a_{ij}\left(1-r_{ij}/r_{c}\right)\;{\hat{r}}_{ij}\;\left(r_{ij}<r_{c}\right)\\ \;0\;\left(r_{ij}\geq r_{c}\right) \end{array}$$

where ${\vec{r}}_{ij}={\vec{r}}_{i}-{\vec{r}}_{j}$, $r_{ij}=\left|{\vec{r}}_{ij}\right|$, ${\hat{r}}_{ij}=\;{\vec{r}}_{ij}/\left|{\vec{r}}_{ij}\right|$, $a_{ij}$ is the maximum repulsion between beads *i* and *j*, $r_{c}$ is the cut-off radius with value 1.0. The ${\vec{F}}_{ij}^{S}$ follows a harmonic potential and is introduced between beads connected by covalent bonds to simulate polymer chains. The dissipation strength is set as 4.5 and random noise strength is 3.0 [35,36].

The model systems consist of block copolymers made of A-, B- and, for one system, C-beads in a solvent S. A block is made of multiple beads of the same chemistry. B-beads are solvophilic. Polymers and the solvent are coarse grained so each DPD bead has a volume of 900 Å^3^. The interaction parameters $a_{ij}$ are chosen so that A-, B- and C-blocks corresponded to poly(ethylethylene), poly(ethylene oxide), and poly(perfluoropropylene oxide) blocks in water S (see Table 1; further details can be found in [37,38]). The interactions between the wall and A-, B-, C- and S-beads are all strongly repulsive. So, the wall is highly solvophobic and the energy cost per bead for polymer-wall and solvent-wall interactions are the same.

The spring constant k for the spring force ${\vec{F}}_{ij}^{S}$ is set at k = 25.0. This allows experimentally observed aggregates of $A_{4}B_{6}C_{2}$ terpolymer in water to be reproduced accurately [37,39] (see Figure S1. n.b. figures and tables denoted with ‘S’ can be found in the supplementary materials available online).

**Figure 1:**
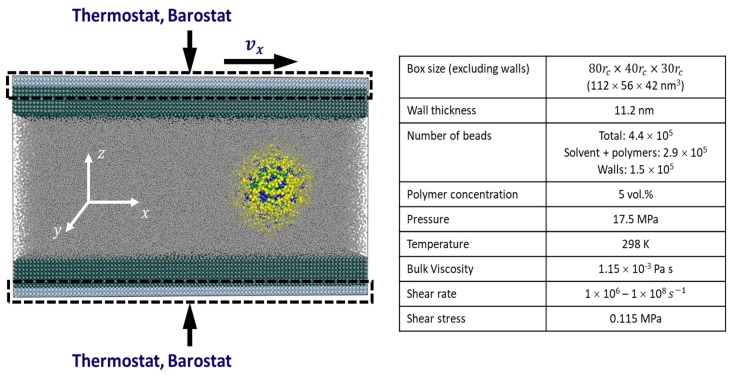
Test conditions of simulations of vesicles under shear.

All simulations are conducted in the NPT conditions at 298 K and 17.5 MPa. The conditions of the simulations and the layout of the computational domain are shown in Figure 1. These conditions are close to those observed in tribological contacts. While the pressure applied in this work is in the low end of those normally encountered in engineering applications, the low pressure-viscosity coefficient of water [40] means that the results are not very sensitive to pressure.

**Figure 2:**
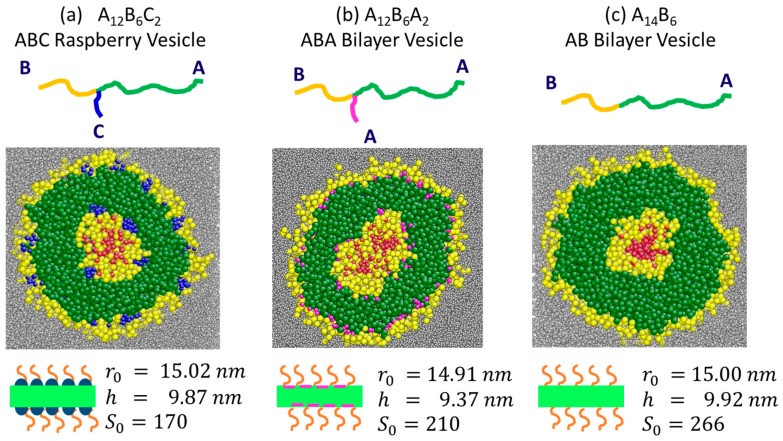
Equilibrium vesicle structures formed by block copolymers (**a**) $A_{12}B_{6}C_{2}$ (ABC); (**b**) $A_{12}B_{6}A_{2}$ (ABA); and (**c**) $A_{14}B_{6}$ (AB). Top row: schematics of the block copolymers and colour codes of individual polymer blocks. Middle row: cross-sections of the vesicles. The red beads are encapsulated S-beads ($S_{o}$) and grey beads are free S-beads ($S_{n}$). Bottom row: schematics highlight the morphology of the vesicle membrane. $r_{0}$ and h are the radius and the membrane thickness of the vesicle (see Figure S2 for definitions). The number of encapsulated $S_{o}$ beads differ among vesicles. Note for (**b**), the long *A*-block and the short *A*-block are green and magenta. They have the same monomer chemistry but differ in block length.

Three block copolymers are studied. They are two star terpolymers-$A_{12}B_{6}C_{2}$, and $A_{12}B_{6}A_{2}$- and one diblock copolymer-$A_{14}B_{6}$ (see schematics in Figure 2). The polymer volume concentration $\varphi_{p}\;=$ 5% is used for all simulations. Initially homogeneous solutions of polymer chains in S-beads are allowed to reach equilibrium in a periodic box of size $80.0r_{c}\times 40.0r_{c}\times 30.0r_{c}$ (112×56×42 nm^3^), as detailed in [38]. All three polymers form vesicles with hollow cores.

Vesicles are exposed to shear both in the bulk and between walls. These two conditions are referred to as bulk shear and wall shear respectively. Bulk shear computations are carried out in a box of size $80.0r_{c}\times 40.0r_{c}\times 30.0r_{c}$. Periodic boundary conditions are applied in the x- and y-directions. In the z-direction, periodicity is modified by altering the momentum of particles across the z-boundary so that a velocity gradient develops.

Wall shear is applied by adding walls to the domain at the top and bottom boundaries (z-direction) as shown in Figure 1. The walls are 11.2 nm (8$r_{c}$) thick and are built with a face centered cubic structure with (001) plane facing the fluid. The lattice spacing is 0.855$r_{c}$. The interaction parameter between wall beads is 18 to ensure the wall is impenetrable. The DPD thermostat and Berendsen barostat [41,42] are applied to the outer layers (about 2.5 nm) of walls (see dash-line rectangles, Figure 1). The desired temperature and pressure are established in approximately after 500,000 time steps, each time step Δt= 0.04, after which a constant velocity in x-direction is applied to the upper wall. A steady state flow profile is typically achieved after 2,000,000 time steps. All results presented are from at least two initialisations and are reproducible.

## 3. Results and Discussions

To facilitate the following discussion, some nomenclature is introduced. The radius of the vesicle $r_{0}$ is defined as the minimum distance between the centre of mass of the aggregate and the nearest solvophilic B-beads in the outer skin of the aggregate. $r_{0}^{\prime }$ defines the cavity of the vesicle and is the maximum distance between the centre of mass of the vesicle and the B-beads forming the inner skin. The thickness of the membrane is $h=\;r_{0}-r_{0}^{\prime }$. The definition of the membrane excludes the solvated layers. S-beads encapsulated by the vesicle before shear is applied are labelled $S_{0}$, all other S-beads are labelled $S_{n}$. Details of definitions of parts of a vesicle and its dimensions are in Figure S2. Unless otherwise stated, the time t=0 is the time when shear is applied.

### 3.1. Characterisation of Vesicle Structure at Equilibrium

The equilibrium structures, size and encapsulation capacity of the vesicles depend on their constituent copolymers. The amount of $S_{0}$ beads encapsulated by ABC, ABA and AB vesicles are 170, 210 and 266 respectively (see Figure 2). In all cases, the inner leaflet of the bilayer membrane is made of solvophobic A-beads (green, Figure 2) while the inner and outer shells of the membrane are solvophilic B-bead skins (yellow, Figure 2) surrounded by solvated B-bead layers (yellow ‘hairs’, Figure 2). Both ABA and AB vesicles are bilayer vesicles (see Figure 2b,c respectively). The difference in polymer chain architecture, causes the ABA vesicle to be slightly smaller (see $r_{0}$ in Figure 2). As a result, the ABA vesicle encapsulates fewer S-beads than the AB vesicle. The ABC vesicle has nanodomains composed of solvophobic C-beads (blue, Figure 2a) on the outer and inner skins of its bilayer membrane, making it a raspberry vesicle [43,44]. The existence of solvophobic domains on the solvophilic surfaces causes the ABC vesicle to encapsulate the least amount of S-beads out of the 3 vesicles investigated.

The effectiveness of S-bead encapsulation of the tested vesicles is investigated by monitoring how the amount of $S_{0}$ changes with time in bulk solutions in the absence of shear, as shown in Figure S3. In 2 μs, only 3 (1%), 4 (2%), 2 (1%) $S_{0}$-beads have leaked out of the AB, ABA and ABC vesicles respectively.

### 3.2. Cargo Release in Bulk Shear Condition

Cargo release under bulk shear is investigated at shear rates $\dot{\gamma }=$ $1\times 10^{7}$ and $1\times 10^{8}{\;s}^{-1}$. In all cases, very few encapsulated $S_{0}$-beads are released, so only results from the highest shear rate $\dot{\gamma }=1\times 10^{8}\;s^{-1}$ are presented. The shear flow profiles presented in Figure S4a show the inclusion of a vesicle does not change the profile, which remains approximately linear.

The shear stress experienced by the vesicles are about 0.1 MPa in all cases. Shear deforms the vesicles by slightly stretching them in the flow direction. The stretching of the vesicles by the shear flow can be detected by comparing the radius $r_{\theta }$ of the vesicles projected on the x-y plane with the radius $r_{\varphi }$ projected in the orthogonal direction (see Figure S2c on definitions of $r_{\theta }$ and $r_{\varphi }$ and Figure S5e–g for values of $r_{\theta }$ and $r_{\varphi }$). As a result of the deformation, the average surface stresses of the membrane are higher in the θ direction than in the φ direction (see Figure S5a–c).

**Figure 3:**
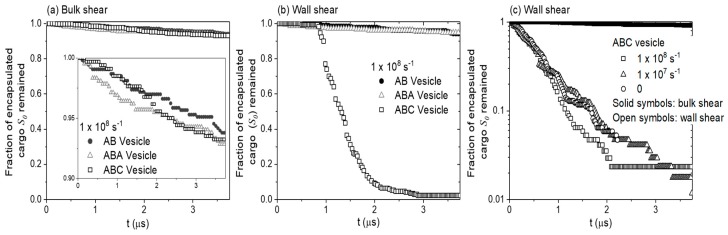
The fraction of encapsulated $S_{0}$-beads remained in a vesicle at shear rate $\dot{\gamma }$ = 1 × 10^8^ $s^{-1}$ under (**a**) bulk shear and (**b**) wall shear; and for ABC vesicles (**c**) at different shear rates. Note (**c**) is in log-linear scale and data represented with open symbols has been offset so time t = 0 corresponds to the time ABC vesicles touch the wall.

$S_{0}$-beads are released from all 3 vesicles under bulk shear in microseconds. In a tribological contact with a diameter in the order $10^{-5}\sim 10^{-3}$ m, and fluid entrainment speed in the order of 1 m/s, a release time of 1 μs is reasonable. The rate of cargo release is examined by monitoring how the fraction of encapsulated $S_{0}$-beads changes with the duration of shear (see Figure 3a). All vesicles have similar cargo release rate, with AB vesicle showing a slightly lower release rate than the other two (see solid circles, insert in Figure 3a). At time t = 2 μs, 3% of $S_{0}$ beads for AB vesicle and 4.5% for ABA and ABC vesicles have been released. Comparing these release rates with those without shear (1, 2, and 1% for AB, ABA and ABC vesicles respectively, Figure S3) shows that bulk shear only marginally facilitates cargo release. After shearing the vesicles for 3.5 μs, about 5.7% of $S_{0}$-beads in AB vesicle and 6.7% in ABC and ABA vesicles are released.

While $S_{0}$-beads are being released, small numbers of $S_{n}$-beads, i.e., S-beads initially outside the vesicles, find their way inside (see Figure S6b). This is not surprising as in this study there is no chemical or geometric difference between $S_{0}$- and $S_{n}$-beads. The amount of $S_{n}$-beads entered the vesicles is comparable to the amount of $S_{0}$ beads released so that the total amount of S-beads inside each vesicle remains relatively constant (see Figure S6c). All 3 vesicles having similar release rate suggests they have similar cargo release mechanisms.

**Figure 4:**
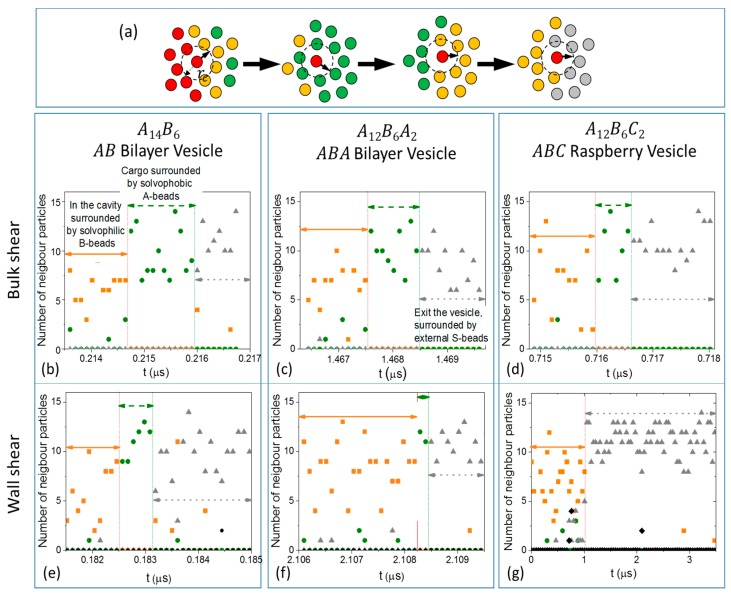
(**a**) Schematic showing the neighbour particles that a tracked encapsulated $S_{o}$-bead (red bead with arrow) encountered when it migrates out of the vesicles. Neighbour particles are particles that locate within distance $r_{c}$ from the centre of mass of the tracked particle. The numbers of A-, B- and S-neighbour particles vs. time for a representative encapsulated $S_{0}$-bead during its release from AB, ABA, and ABC vesicles under (**b**–**d**) bulk shear and (**e**–**g**) wall shear. Solid yellow, dashed green, and dotted grey arrows are the solvophilic B-bead region, solvophobic A-bead region, and the solvent region. For (**b**–**f**), the solid yellow arrow corresponds to regions of the inner shell and the dashed green arrow the A-bead inner leaflet of the vesicle membrane. For (**g**), the solid yellow arrow corresponds to the solvophilic pathway.

To gain insights into the cargo release mechanisms of the three vesicles, positions of individual $S_{0}$-beads are tracked during their release. At every time step, the identities of beads surrounding the tracked $S_{0}$-beads are recorded. At any time t, a bead X is considered a neighbour of a tracked $S_{0}$-bead Y if the distance between X and Y is less or equal $r_{c}$ (see Figure 4a). As a $S_{0}$ bead moves, the composition of its neighbourhood changes (see Figure 4a). Thus one can count the number of A-, B-, S- and C-neighbours (if any) of a $S_{0}$-bead at a given time t. The composition of the neighbourhood crossed by a $S_{0}$-bead reveals the path it follows during its release. Typical results from $S_{0}$-beads released from AB, ABA, and ABC vesicles under bulk shear are shown in Figure 4b–d respectively. Focusing on the AB vesicle (Figure 4b), the released $S_{0}$-bead is initially surrounded mainly by B-beads (yellow) with few A-beads (green) (solid yellow arrow region, Figure 4b). The bead then crosses a region made of A-beads only (green dash arrow region, Figure 4b) and finally enters a region composed mainly of $S_{n}$-beads (grey) (grey dotted arrow region, Figure 4b). All released $S_{0}$-beads from all three vesicles experience similar environments in the same sequence, although the time spent in each region may differ (see Figure 4c,d for ABA and ABC vesicles respectively). These results indicate that when an encapsulated molecule $S_{0}$-bead is being released, it travels through the membrane.

**Figure 5:**
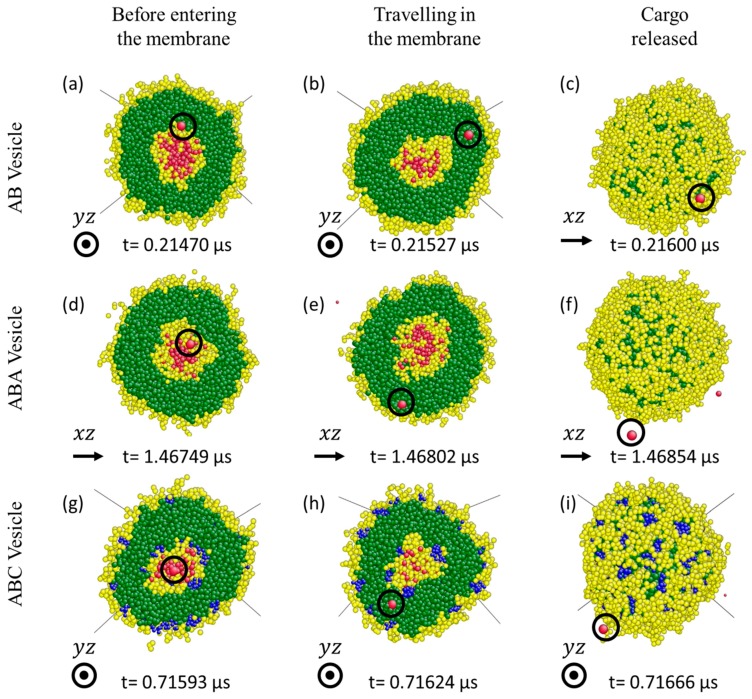
Snapshots showing a representative encapsulated $S_{0}$-bead (circled) before it enters the membrane, in the A-bead inner leaflet and out of the vesicle for (**a**–**c**) AB; (**d**–**f**) ABA; and (**g**–**i**) ABC vesicles. They are under bulk shear, shear rate = 1 × 10^8^ $s^{-1}$. Shear is applied along x direction from left to right (see arrows). The dot in the circle means shear is applied outwardly from the paper.

The initial B-bead environment (solid yellow arrow region, Figure 4) encountered by the tracked $S_{0}$-bead is the solvated B-bead layer in the cavity of the vesicle as the inner shell of the membrane is made of mainly solvophilic (and solvated) B-beads. The A-bead region (green dash arrow region, Figure 4) that follows shows that the $S_{0}$-bead goes through the inner leaflet of the vesicle membrane. Finally, the bead is released and is surrounded by $S_{n}$-beads (grey dotted arrow region, Figure 4), the solvent outside of the vesicles. Snapshots presented in Figure 5 show examples of released $S_{o}$-beads (red beads highlighted with black circles) before entering the membrane, in the membrane and just outside of the vesicle confirm that released $S_{o}$-beads have to travel through the A-bead inner leaflets (green) of the membrane. Analysis of the coordination numbers of A-beads shows that the structure of the membrane is not significantly affected by shear (See Figure S7). Furthermore, analysis of the membrane density shows no correlation between the passage of $S_{0}$-beads being released and the local density fluctuations (See Figure S8). These findings exclude the formation of pores as release mechanism.

**Figure 6:**
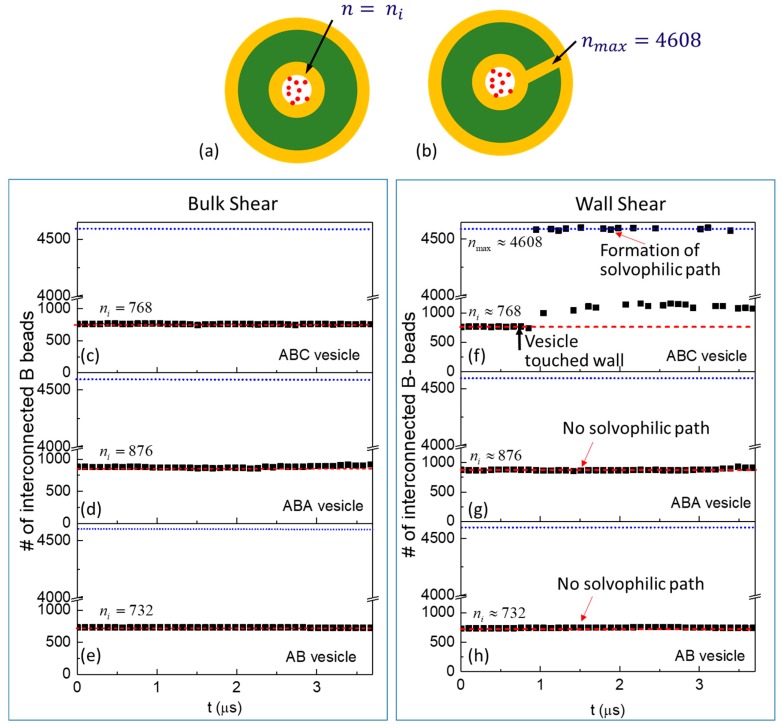
Schematics showing for a generic vesicle (**a**) no connection between the inner and outer B-bead shells and $n=n_{i}$ is defined as the number of interconnected B-beads that constitute the inner shell at static conditions and (**b**) the inner and outer B-bead shells are linked. As a result the number of interconnected B-beads increases to $n=n_{max}$. For (**a**,**b**), yellow regions are solvophilic B-bead region, i.e., the inner and outer shells; while the green region is the solvophobic A-bead region, i.e., the inner leaflet of a membrane. Note hydrated layers and any solvophobic domains exist on the inner and outer shells are omitted. Number of interconnected B-beads starting from the inner shells for vesicles (**c**–**e**) under bulk shear, and (**f**–**h**) under wall shear. Red dash and blue dotted lines shows the possible minimum $n_{i}$ and maximum $n_{max}$ number of interconnected B-beads in the vesicle.

The topology of the vesicle membranes can also be monitored to probe the release mechanism under bulk shear. This involves counting the number of interconnected B-beads from the inner shell (see Figure 6). Before shear is applied, the number of interconnected B-beads in the inner shell n is $n=n_{i}$ (Figure 6a). A change in membrane topology changes the number of interconnected B-beads. The maximum number of interconnected B-beads $n_{max}$ is reached when the inner and outer B-bead shells are linked (Figure 6b). The numbers of interconnected B-beads are presented in Figure 6c–e as functions of time when bulk shear is applied to ABC, ABA, and AB vesicles respectively. In all cases, n remains constant and is equal to $n_{i}$ thus confirming that no pathway through the thickness of the membrane for the release of $S_{0}$-beads has been created. This explains the slow release of $S_{0}$-beads: it is energetically unfavourable for $S_{0}$-beads to enter the A-bead inner leaflet and a significant fraction of the $S_{0}$-beads entering the A-bead layer return back into the vesicle core very quickly. (See Figure S10).

**Figure 7:**
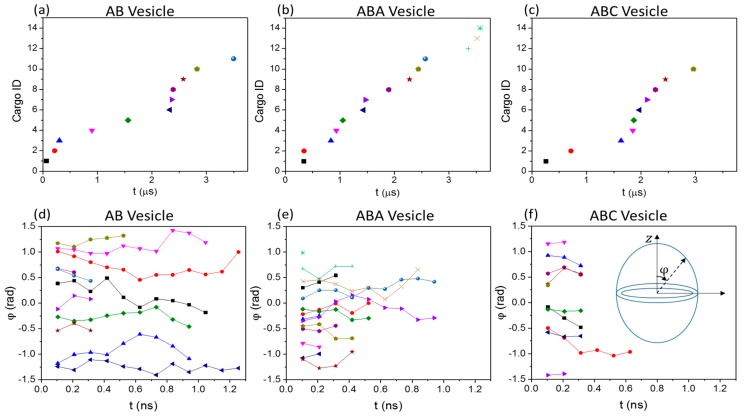
(**a**–**c**) The time at which $S_{0}$-beads (which are subsequently released) enter vesicle membranes under bulk shear. Each individual symbol corresponds to one released $S_{0}$-bead. (**d**–**f**) The direction at which released $S_{0}$-beads taken during their release under bulk shear. Tracks with the same symbols as those from (**a**–**c**) of the same vesicle are from the same released beads. The y-axis corresponds to the angle of the trajectory of $S_{0}$-beads with respective to the z-direction (see insert in (**f**)).

Without any specific pathway for the release of $S_{0}$-beads, the question then is whether preferential locations exist in the membrane for cargo release. The $S_{0}$-bead release events are spread across the duration of the simulations (Figure 7a–c) and are rare events. Figure 7d–f show how the angles φ between the release paths and the z direction changes with time for all released $S_{0}$-beads from the three vesicles. For all three vesicles, $S_{0}$-beads are released at various φ ranging from −π/2 to π/2, suggesting there is no preferential location on the membrane for cargo release. This supports results in Figures S6 and S7 that the positions of cargo release do not have a strong correlation with the local particle density of the membrane. Furthermore, φ remains relatively constant for each path showing that $S_{0}$-beads travel radially through the membrane during release.

The time taken by released $S_{0}$-beads to cross the membranes are about 0.3 ns for ABC vesicles, 0.4 ns for ABA and 1 ns for AB vesicles. Membrane thicknesses of these vesicles are comparable (9.92, 9.37 and 9.87 nm, respectively) but membrane density (Figure S8) and A-bead coordination numbers (Figure S7) are different and therefore the travel times are ascribable to differences in membrane properties. It should also point out that travel times are very small compared to the average interval between release events, which take place every few 100 ns.

### 3.3. Cargo Release under Wall Shear

In the previous section, we show that ABC, ABA and AB vesicles behave similarly when subjected to bulk shear. In this section, we present results obtained when these vesicles are being sheared between two walls under the conditions stated in Figure 1. Tests were conducted at $\dot{\gamma }=0,\;1\times 10^{7}$ and $1\times 10^{8}$ s^−1^. As shown in Figure 3c, shear rate has marginal effect on the cargo release under wall shear, hence results from shear rate $\dot{\gamma }=1\times 10^{8}$ s^−1^ are presented.

The rate of release of encapsulated $S_{0}$-beads when the three vesicles are sheared between two walls are shown in Figure 3b. It is clear that ABC vesicle (squares) behaves very differently from both ABA (triangles) and AB (circles) vesicles. Shearing AB and ABA vesicles between walls give similar $S_{0}$-bead release rates to those observed in bulk shear (Figure 3a), with both the release of $S_{0}$-beads (Figure 3, triangles and circles) and the intake of $S_{n}$-beads (Figure S6e, triangles and circles) being very slow. As a result, the total number of S-beads (Figure S6f, triangles and circles) in the core of the vesicle remains relatively constant. Examining the constituents of neighbouring particles encountered by released $S_{0}$-beads in these two vesicles (Figure 4e,f) shows features similar to those observed when the vesicles are under bulk shear (Figure 4a,b). This shows that the $S_{0}$-bead release mechanisms for ABA and AB vesicles under wall shear remain the same as those under bulk shear: the encapsulated $S_{0}$-beads are released through the membrane and no significant changes in the structure of the membrane accompany the process. This is confirmed by examining the topology of the membranes of these vesicles, showing the same signature as that observed under bulk shear, with the number of interconnected B-beads being constant and is $n_{i}$ (Figure 6g,h).

Examining the rate of cargo release from the ABC vesicle when sheared between walls shows two distinct regions (open squares, Figure 3b). For t<1 μs, ABC vesicle shows a similar release rate to that observed in bulk shear. At this slow release stage, the released $S_{0}$-beads first enter a solvophilic B-bead zone (inner shell, yellow solid arrow, Figure S9). The $S_{0}$-beads then proceed to the solvophobic A-bead zone (inner leaflet, green dash arrow, Figure S9), until they are finally released and surrounded by external $S_{n}$-beads (exterior of the vesicle, grey dotted arrow, Figure S9). This indicates that the cargo release mechanism for ABC vesicle at this slow release stage is similar to those of ABA and AB vesicles. While $S_{0}$-beads are released, $S_{n}$-beads enter the vesicles slowly (Figure S6e, circles). At this stage, the total number of encapsulated S-bead is relatively constant over time (Figure S6f, circles).

**Figure 8:**
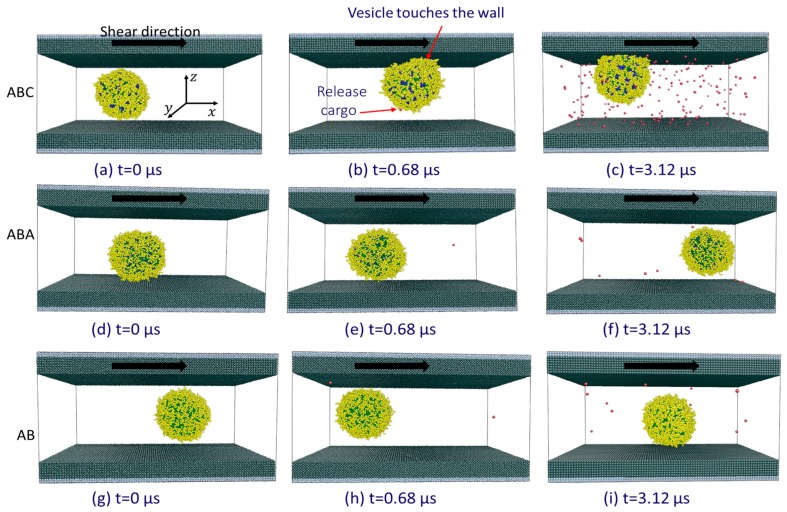
Snapshots of (**a**–**c**) ABC, (**d**–**f**) ABA and (**g**–**i**) AB vesicles being sheared between walls, shear rate = 1 × 10^8^ $s^{-1}$. The red beads are $S_{0}$ beads released. Solvent beads are omitted for clarity. Arrows on the shearing wall show the shear direction.

At t ~ 1 μs, there is a sudden increase in cargo release rate, as observed by a rapid drop in the fraction of $S_{0}$-beads remaining in ABC vesicle (Figure 3b). Most of these fast released $S_{0}$-beads are first surrounded by solvophilic B-beads (yellow) then by $S_{n}$-beads (grey) outside of the vesicle (see Figure 4g). Thus, unlike their slow-release counterparts in AB, ABA and ABC vesicles, fast-release $S_{0}$-beads from ABC vesicle encounter very few, if any, A-beads in their release paths. This is counter-intuitive as the majority of the vesicle mass is A-beads. At this stage, the intake rate of $S_{n}$-beads is also high. Indeed the amount of $S_{n}$ beads intake (Figure S6e, circles) outweighs the amount of $S_{0}$-beads released. As a result, the total number of S-beads in ABC vesicles increases with time during wall shear (Figure S6f, circles). The rate of increase however is decreasing and then stabilises. All $S_{0}$-beads are released by t= 3 μs while $S_{n}$-beads continue to enter the vesicles. All these observations suggest that unique release mechanisms are in operation at the fast release stage. Interestingly the sudden change in cargo release rate in ABC vesicle occurs shortly after the vesicle touches the wall (see Figure 8b). While ABC vesicle reaches the wall at different times in different simulation runs (with the same and different shear rates), the behaviour of the fast cargo release remains the same (Figure 3c). This shows that for ABC vesicle, the critical event for fast cargo release is the contact between the vesicle and the wall.

No wall touching event is observed for AB vesicles (see Figure 8g–i). ABA vesicles only touch the wall in longer simulation runs (see Figure 8d–f) and the results will be discussed in Section 3.3.2 on the significance of the shearing wall.

#### 3.3.1. Formation of Solvophilic Pathway in *ABC* Vesicles

**Figure 9:**
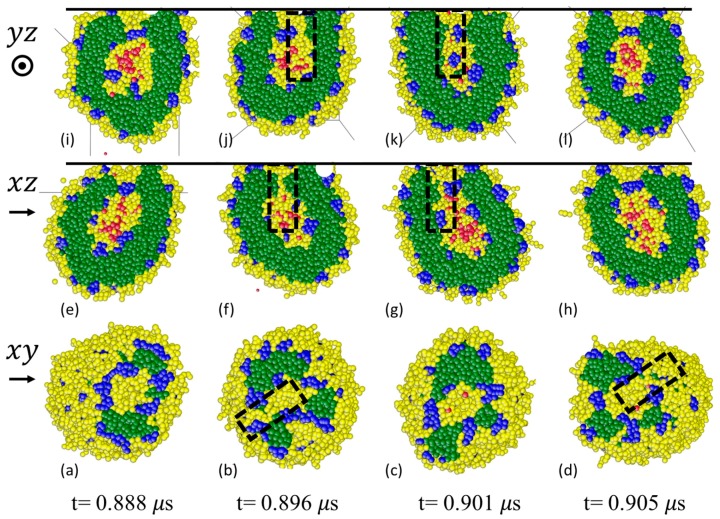
Snapshots showing the formation of solvophilic paths after the ABC vesicle touches the wall. The vesicle is under wall shear at shear rate = 1 × 10^8^ $s^{-1}$. (**a**–**d**) are cross-sections at the vesicle-wall interface (x-y plane); (**e**–**h**) cross-sections on x-z plane; and (**i**–**l**) cross-sections on y-z plane. The thick black solid lines in (**e**–**l**) denote the wall. The dashed rectangles highlight the solvophilic paths. Note the dynamic nature of the path. Shear is applied along x-direction from left to right (see arrows). The dot in the circle means shear is applied outwardly from the paper.

Counting the number of interconnected B-beads for the ABC vesicles $n_{ABC}$ shows that before contact with the wall, there are $n_{ABC}$ = 768 (Figure 6f). This is equal to the total number of B-beads on the inner shell before shear $n_{i}$. After contact with the wall, $n_{ABC}$ is much larger than $n_{i}$ intermittently (Figure 6f). For a significant amount of time $n_{ABC}$ is at its maximum $n_{max}$ = 4608 which is only possible when the inner and outer B-bead shells are connected. At these occasions, a solvophilic path from the cavity to the exterior of the vesicle is formed and acts as a preferential pathway for the fast release of $S_{0}$-beads. This is confirmed by snapshots of cross-sections of ABC vesicle after it has touched the wall, see Figure 9. The cross-sections on the x-y plane (Figure 9a–d) show the interface between the vesicle and the wall while those on the x-z (Figure 9e–h) and y-z (Figure 9i–l) planes are orthogonal to the interface. When the vesicle touches the wall, a solvophilic zone made of B-beads (yellow) forms at the vesicle-wall interface near the center of the contact (Figure 9a). The interfacial solvophilic zone is surrounded by A-beads (green) which are also in contact with the wall. The contact line among the wall, the vesicle and the solvent is made of B-beads which are part of the vesicle outer shell. In some locations, the interfacial solvophilic zone and the contact line are bridged by C-domains (blue). At t = 0.888 μs, $S_{o}$-beads (red) are contained in the vesicle cavity, separated from the newly formed interfacial solvophilic zone by a plug made of C-beads (blue) (see Figure 9e,i). The morphology of the interfacial region changes with time. At t = 0.896 μs, the interfacial solvophilic zone is connected with the outer solvophilic shell (highlighted with a rectangle in Figure 9b). At the same time, a solvophilic passage has formed between the cavity and the interfacial solvophilic zone (highlighted with rectangles in Figure 9f,j), so a continuous solvophilic pathway that connects the cavity of the vesicle to the outer shell is established. This pathway can act as a highway for cargo release as it screens $S_{0}$-beads from contacting A-beads during their release. Interestingly, there is no $S_{0}$-bead at the interfacial solvophilic zone at this particular time. This can be due to the dynamic nature of the pathways. These solvophilic connections are temporary and can form in various locations (compare t = 0.896 with t = 0.901 and t = 0.905 μs in Figure 9). In addition, a partial pathway can form. For example, at t = 0.901 μs a solvophilic channel is formed between the inner shell and the interfacial solvophilic region (see dash boxes in Figure 9g,k) such that $S_{o}$-beads can travel through this channel to the interfacial solvophilic zone (see red beads in Figure 9c). These $S_{o}$-beads however are trapped because C-domains (blue) have blocked their way out of the vesicle. At t = 0.905 μs, while the solvophilic passage between the cavity and the interfacial solvophilic zone is closed, a solvophilic pathway is available between the interfacial solvophilic zone and the outer shell (dash box in Figure 9d). As a result the trapped $S_{o}$-beads shown in Figure 9c move out of the vesicle and are thus released. This highlights the release process can potentially be 2-step, as well as a 1-step process.

The likelihood of the formation of a solvophilic pathway in ABC vesicle is revealed in Figure 6f, which shows that the number of interconnected B-beads in the inner shell are almost always above 768 after the vesicle touches the wall, suggests a partial hydrophilic pathway is almost always available. The formation of a partial pathway means cargo release take place in two steps. Occasionally the number of interconnected *B*-beads reaches 4608, indicating the formation of a complete path. These results highlight the dynamics nature of pathways promoting the release of $S_{o}$-beads.

#### 3.3.2. The Significance of the Shearing Wall

Cargo in ABC vesicle is released through the membrane slowly under bulk shear, and through solvophilic pathways at a much higher rate with wall shear once the vesicle touches the wall. The shear rate only has a marginally effect (Figure 3c). The effect of the existence of a shearing wall on the release mechanism of ABC vesicle is unexpected. Why solvophilic pathways form in ABC vesicles after they reach the wall?

Recall that in this study all beads have the same repulsive interactions with the wall ($a_{AW}=a_{BW}=a_{CW}=a_{SW}=$ 200). B-beads are solvophilic ($a_{BS}=$ 26), while A- and C-beads are solvophobic ($a_{AS}=$ 97, $a_{CS}=$ 125). Thus there is a substantial energy cost for the vesicle to be in contact with the wall. However if the vesicle is in contact with the wall, the solvent-vesicle interface and the solvent-wall interface are removed in exchange for the generation of a vesicle-wall interface. That would lead to a reduction of overall potential energy of the system.

**Figure 10:**
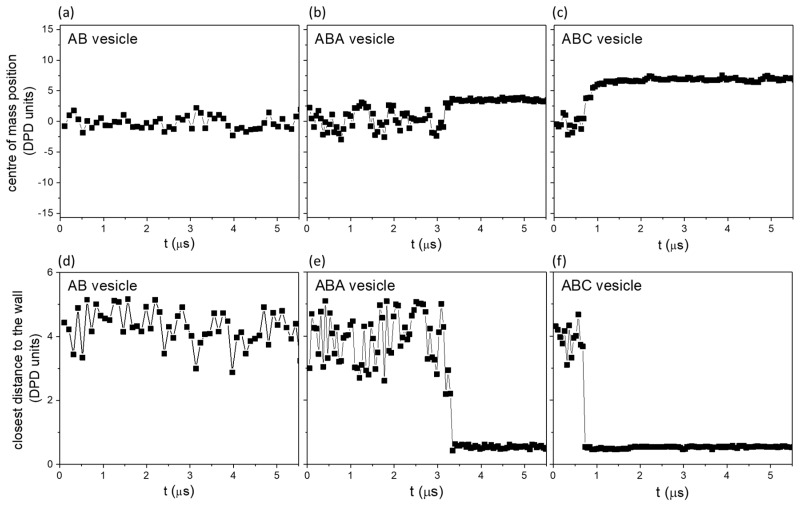
(**a**–**c**) The separations between centre of mass of vesicles and the wall; (**d**–**f**) The closest distance between the vesicle and the wall, defined as the closest distance between beads on the outer skin (excluding the solvated layer) and the wall.

The above discussion should apply to all three vesicles in this study. For AB vesicles, the position of the centre of mass of the vesicle is about zero (equi-distance from the two walls) throughout the simulation run (Figure 10a). The closest distance between the wall and the vesicle remains about 4.5 DPD units (see Figure 10d). For a vesicle to touch the wall, the S-beads between the vesicle and the wall must be removed. In the case of AB vesicle, the outer shell is mainly B-beads although some A-beads at the proximity of the outer shell are also exposed to the solvent due to a shortage of B-beads (as there are more A-beads than B-beads in the system. This has been observed with ABC vesicles [38]). The favourable interactions between B-beads at the outer shell and S-beads make it difficult to remove the S-beads solvating AB vesicles. Thus the contact between AB vesicle and the wall is prevented.

For ABC vesicle, the strong repulsive interaction between C- and S-beads means the vesicle may be less solvated, making the removal of S-beads between the vesicle and the wall easier as compared to AB vesicles. Furthermore, the contact between the ABC vesicle and the wall removes some of the energetically unfavourable C-S contacts. This allows the ABC vesicle to approach more closely and subsequently contact the wall (Figure 10c,f).

**Figure 11:**
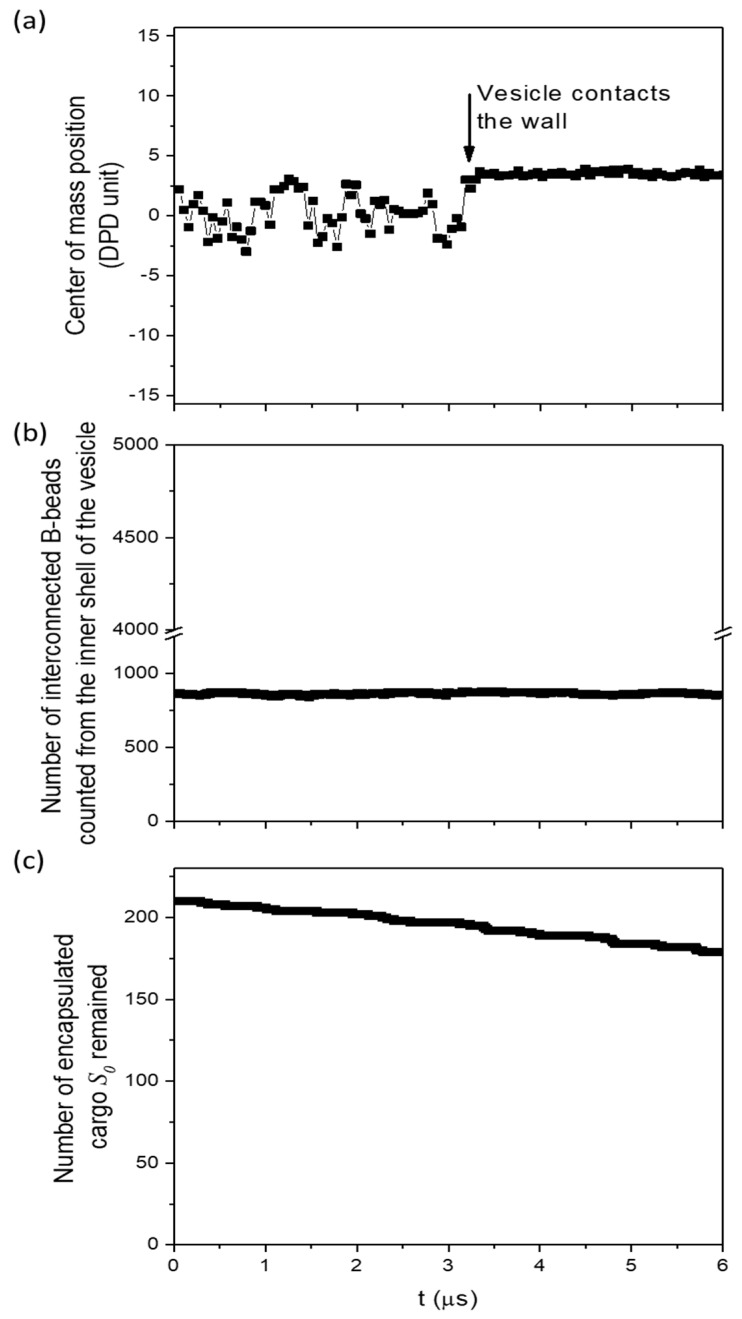
For ABA vesicle under wall shear at shear rate $\dot{\gamma }=1\times 10^{8}$ s^−1^: (**a**) the separation between the centre of mass of the vesicle and its nearest wall; (**b**) the number of inter-connected B-beads counted from the inner shell, with an increase signified the formation of solvophilic pathway; (**c**) the amount of encapsulated $S_{0}$ remained.

The ABA vesicle has A-bead nano-domains on its outer shell, in addition to the exposed A-beads underneath solvated B-beads. This makes the interactions between the outer shell of ABA vesicle and the solvent slightly less and more favourable compared to that of AB and ABC vesicles respectively. One may then expect ABA vesicle to take longer than ABC vesicle to touch the wall. This is confirmed with longer simulation runs, where a sudden change and subsequent stabilization of the position of the centre of mass of the vesicle, showing that ABA vesicle has reached the wall (Figure 10b,e). The time when the ABA vesicle touches the wall differs among simulations with different initialization states. The cargo release mechanisms of ABA vesicle is not affected by contact with the wall (Figure 11). For ABC vesicle, the transition from slow to fast release mechanism occurs soon after the vesicle touches the wall (see Figure 3b) and most of the encapsulated $S_{0}$-beads are released in 1 μs. For ABA vesicle, on the other hand, the release rate, even 2.5 μs after the vesicle touches the wall, is similar to that before the vesicle touches the wall (Figure 11c). The number of interconnected B-beads counted from the inner shell of ABA vesicle before and after the vesicle touches the well is the same (Figure 11b) and is equal to the number before the vesicle is sheared. This means that unlike ABC vesicle, no solvophilic pathway is found in ABA vesicle before and after the vesicle touches the wall. As a result, no fast cargo release is observed.

If both ABA and ABC vesicles touch the wall, why solvophilic pathways do not form in ABA vesicle? Compare Figure 10c and Figure 11a, the centre of mass of ABA vesicle (≈15 − 3.5 = 11. 5 DPD units) is further away from the wall than that of ABC vesicle (≈15 − 7 = 8 DPD units). A larger distance of the centre of mass of ABA vesicle from the wall makes it more difficult for B-beads in the inner skin to interact with the wall and form a solvophilic path. In the case of ABC vesicles, A-, B- and C-beads are immiscible (see Table 1) and all have the same interaction energy with the wall. Therefore rearrangement results in the exposure of A-beads to the wall (see Figure 9a) while some of B-beads that belong to the part of the outer shell that touches the wall either move to the contact line (reducing the energy because $a_{BS}=$ 26 < $a_{AB}=$ 38.5 < $a_{BW}=200$) or become part of the interfacial solvophilic zone. The rearrangement reduces the distance between the inner shell and the interfacial zone, and that between the interfacial zone and the outer wall. This promotes the formation of solvophilic paths.

The findings that formation of solvophilic pathways in ABC vesicle under wall shear for cargo release only occurs when the vesicle touches the wall highlights that using polymersomes is a promising strategy for target additives delivery for lubrication. One can imagine that additive encapsulated vesicles can be added in bulk lubricants and these additives are only released at rubbing contacts where separations between rubbing walls are very small. This will reduce the use of additives while potentially increasing their effectiveness.

## 4. Conclusions

Polymeric vesicles are promising candidates as nano-cargo carriers. However, an understanding of cargo release mechanisms is lacking. In this work we focus on how we can use these vesicles in engineering applications where cargo should be released at specific locations.

Vesicles made of three different polymer architectures, ABC, ABA and AB, experience bulk shear and shear between walls. ABC and ABA are star terpolymers and AB is linear block copolymer. For all three vesicles, cargo releases through the vesicle membranes under bulk shear very slowly. In the case of shear between walls, depending on the polymers, two behaviours are observed. For the case of vesicles made of ABA and AB polymers, there is no change in release mechanism as compared to cases of bulk shear. For ABC vesicle, however, a much higher release rate is observed once the vesicle contacts the wall. In this fast release mode, solvophilic paths are formed which allow cargo to travel from the cavity to the exterior of the vesicle quickly. While ABA vesicle also touches the wall, only the ABC vesicle approaches the wall close enough and makes a large contact region with the wall due to the existences of solvophobic C-domains on its surfaces. The contact of the wall drives the rearrangement of the polymer chains and promotes the formation of solvophilic pathways. This leads to fast cargo release. Note the effect of shear rate is marginally in this study.

The effect on the polymer architecture, specifically the use of solvophobic block, to control cargo release is an interesting option. In this work, the solvophobic block in the ABC vesicle allows precise targeting (the wall) and high release rate of cargo to be achieved. This makes ABC vesicle a very attractive option if one wants to delivery surface active ingredients (for example friction modifiers) to specific locations.

## Figures and Tables

**Table 1:** Interaction energy parameters.

Interaction Parameter, *a_ij_* (in DPD Units)	*A*	*B*	*C*	*S* (Water)	*W* (Wall)
***A***	25.0				
***B***	38.5	25.0			
***C***	78.0	89.4	25.0		
***S*** (water)	97.9	26.0	125.0	25.0	
***W*** (Wall)	200	200	200	200	18
Number of monomer in one coarse-grained bead (900 Å^3^)	7.3	14	6	30	

## Supplementary Materials

The following are available online at http://www.mdpi.com/2073-4360/10/3/336/s1, Figure S1: Nanostructured vesicle self-assembled from $A_{4}B_{6}C_{2}$ star terpolymer replicating structures observed experimentally; Figure S2: Definitions of (a) various parts and dimensions of vesicles; (b) definition of azimuth θ and ($\frac{\pi }{2}-$ altitude) Ψ; and (c) projections of radius of a vesicle, $r_{\theta }$ and $r_{\varphi }$; Figure S3: Cargo released from vesicles in bulk solutions in static conditions; Figure S4: Velocity profiles resulted from the applied shear (shear rate $\dot{\gamma }=1\times 10^{8}$ s^−1^); Figure S5: The surface stresses and radii of the cross-sections of the vesicles in the θ- and φ*-*directions; Figure S6: Cargo release and solvent uptake by vesicles under shear; Figure S7: The coordination number of A-beads in the inner leaflets of vesicles under static and bulk shear conditions; Figure S8: particle density distribution on the membrane surfaces while the vesicles experiencing bulk shear; Figure S9: The numbers of A-, B- and S-neighbour particles vs. time for a representative encapsulated $S_{0}$-beads exhibiting slow release from the ABC vesicle under wall shear; Figure S10: The numbers of A-, B- and S-neighbour particles vs. time of representative $S_{0}$-beads which enter the membrane briefly and then returns to cavity for (a) and (b) ABA and (c) and (d) AB vesicles under wall shear.
