# Supplementary material for: Cargo Release from Polymeric Vesicles under Shear
Source: Polymers (Basel). 2018 Mar 19;10(3):336. doi: 10.3390/polym10030336 (PMC6414962; doi:10.3390/polym10030336)
Supplement: Supplementary file 1 [file polymers-10-00336-s001.pdf]

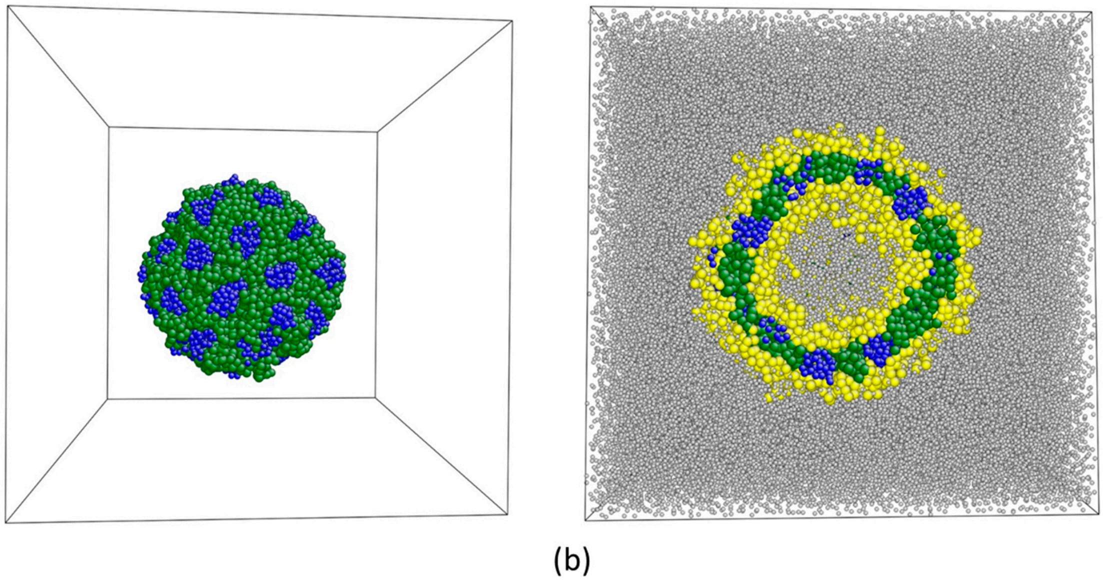

Figure S1. Nanostructured vesicle self-assembled from $A_4B_6C_2$ star terpolymer replicating structures observed experimentally in [1]. Colour: Block A, green; Block B, yellow; Block C, blue; Solvent, light grey. (**a**) morphology of the vesicle. Note solvent and block B are omitted to show clearly that morphology of the vesicle surface; (**b**) cross-section of the vesicle.

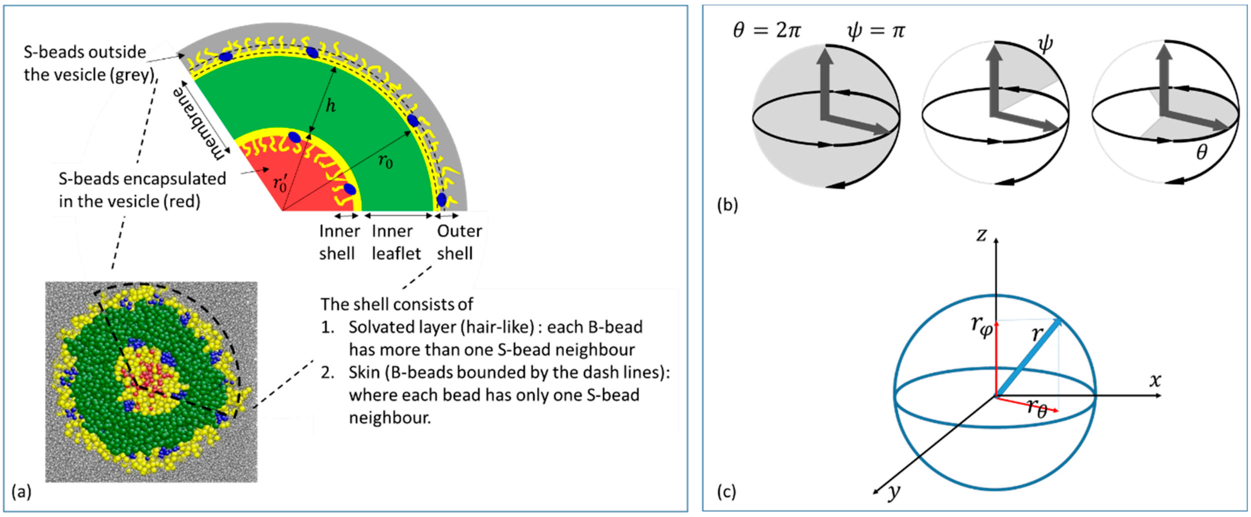

Figure S2. Definitions of (a) various parts and dimensions of vesicles; (b) definition of azimuth θ and ($\frac{\pi}{2}$ – altitude) ψ; and (c) projections of radius of a vesicle, r_θ and r_φ.

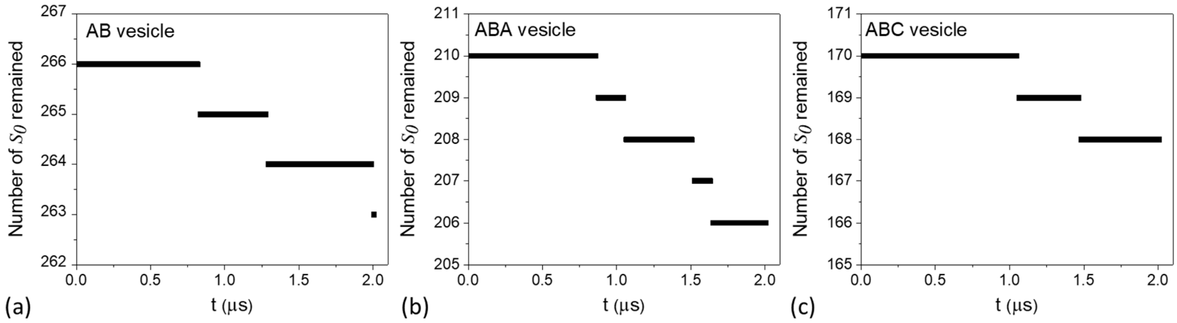

Figure S3. Cargo released from vesicles in bulk solutions in static conditions.

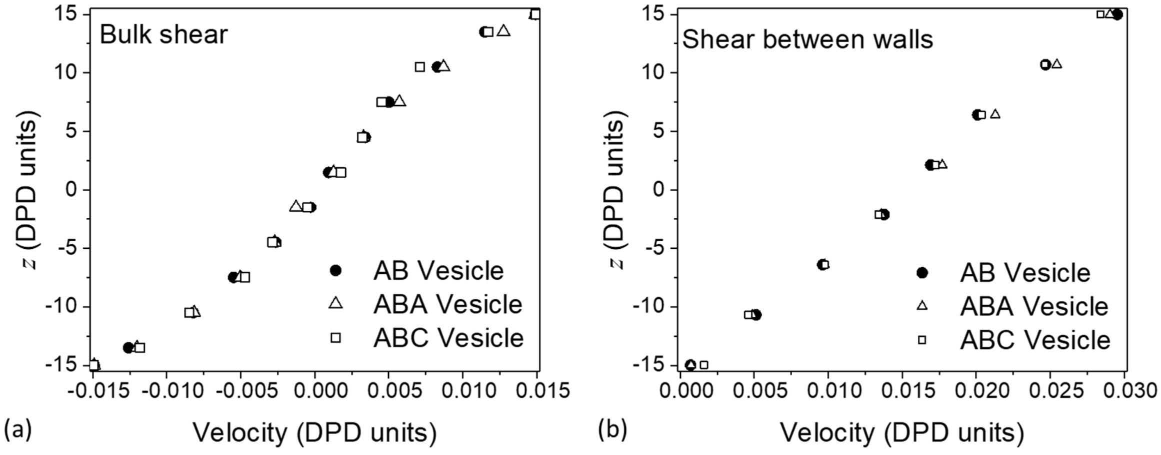

Figure S4. Velocity profiles resulted from the applied shear (shear rate $\dot{\gamma} = 1 \times 10^8$ s^{-1}).

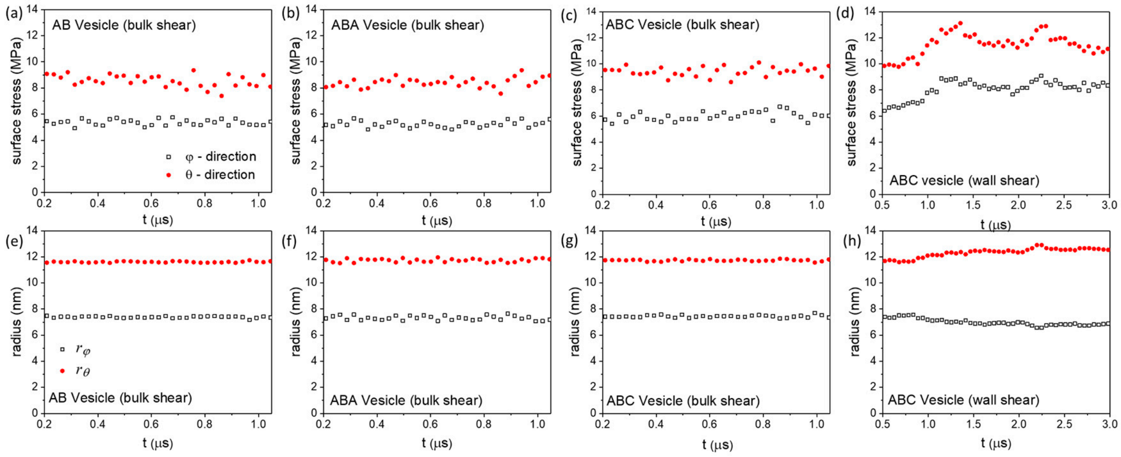

Figure S5. The surface stresses and radii of the cross-sections of the vesicles in the θ- and ϕ-directions, r_θ and r_φ (see Figure S1 for definitions) during bulk shear for AB vesicle: (a) and (e); ABA vesicle: (b) and (f); and ABC vesicle: (c) and (g); and during wall shear for the ABC vesicle: (d) and (h).

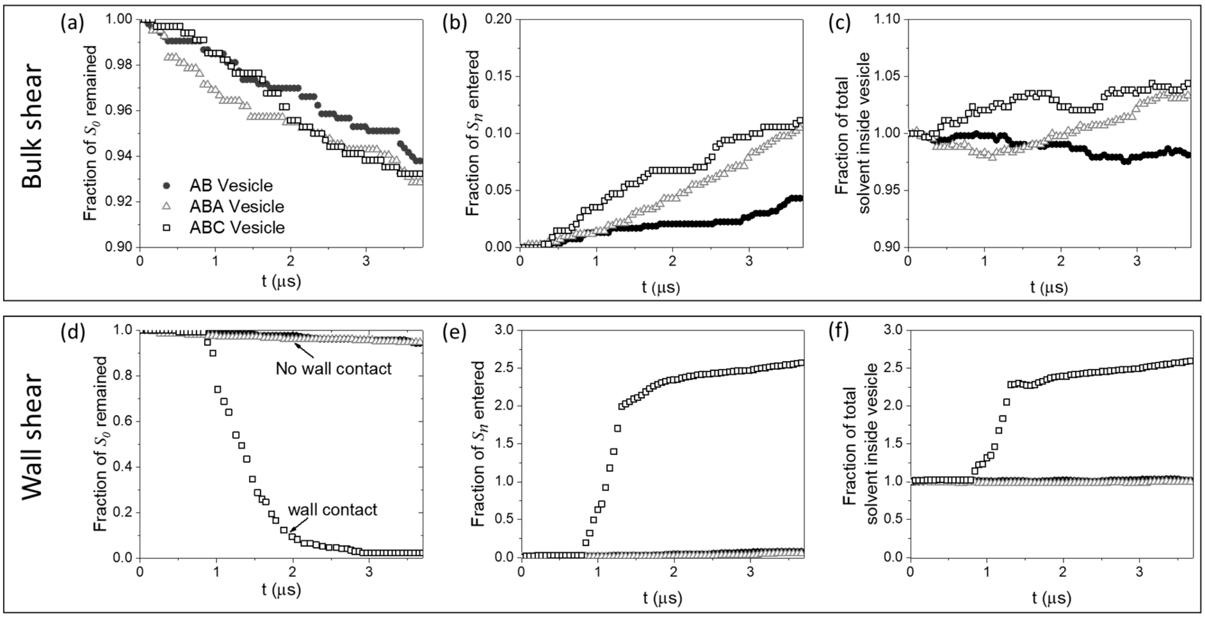

Figure S6. Cargo release and solvent uptake by vesicles under (a) – (c) bulk shear; (d) – (f) wall shear. Shear rate is $1 \times 10^8\ s^{-1}$.

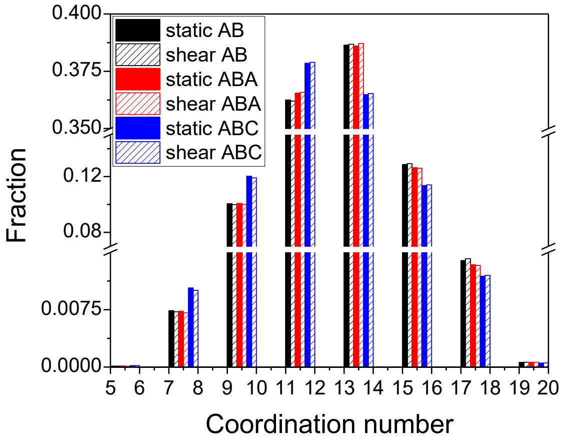

Figure S7. The coordination number of *A*-beads in the inner leaflets of vesicles under static and bulk shear conditions. In all cases, the coordination number distribution, hence average particle packing density of the membrane is not affected by bulk shear.

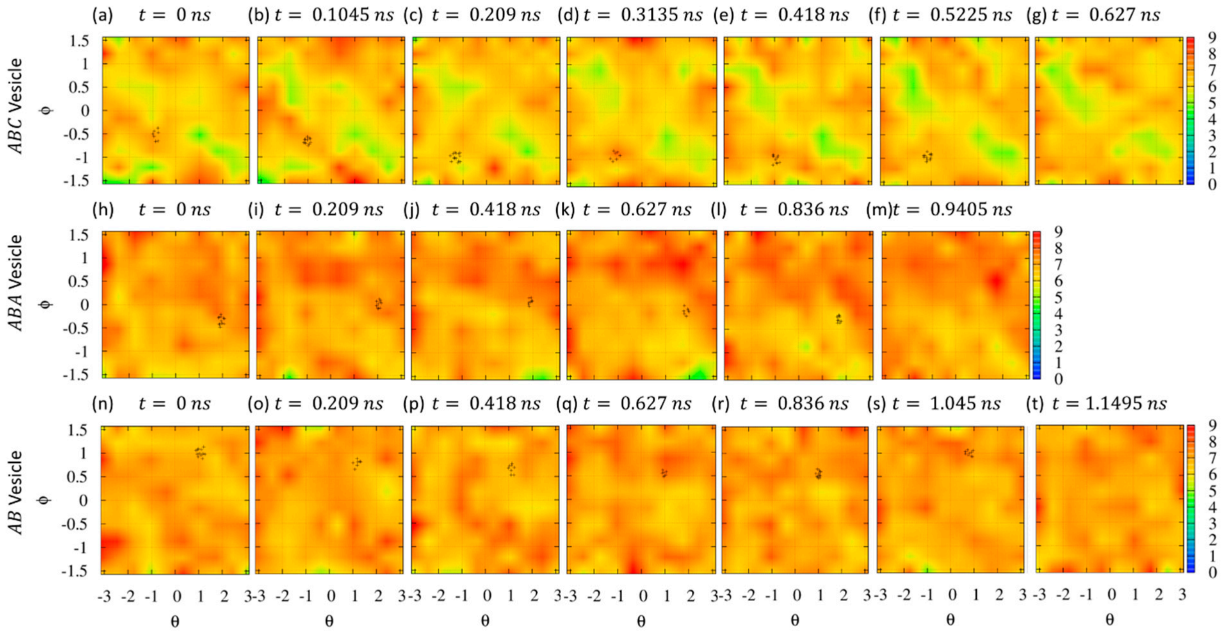

Figure S8. particle density distribution on the membrane surfaces while the vesicles experiencing bulk shear. The black '+' are the beads surrounding a tracked S_0-bead as it travels across the membrane. (The average neighbour number of A-beads in the membrane is 12 in all cases, see Figure S7). Note in all cases, there is no evidence that the tracked S_0 bead travels at locations with lower particle density, i.e. pores.

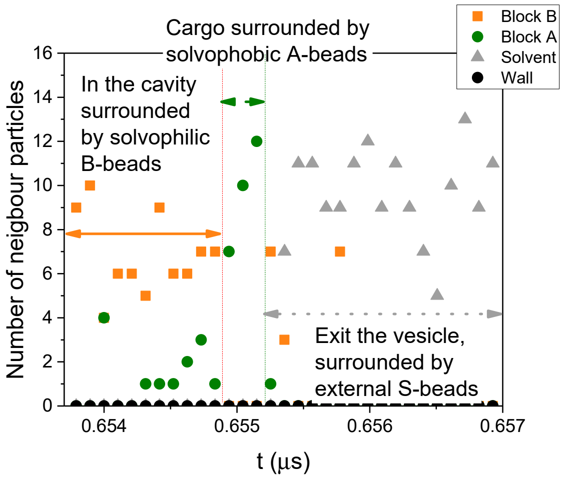

Figure S9. The numbers of A-, B- and S- neighbour particles vs. time for a representative encapsulated S_0-beads exhibiting slow release from the ABC vesicle under wall shear. The slow release process under wall shear is similar to what is observed during bulk shear.

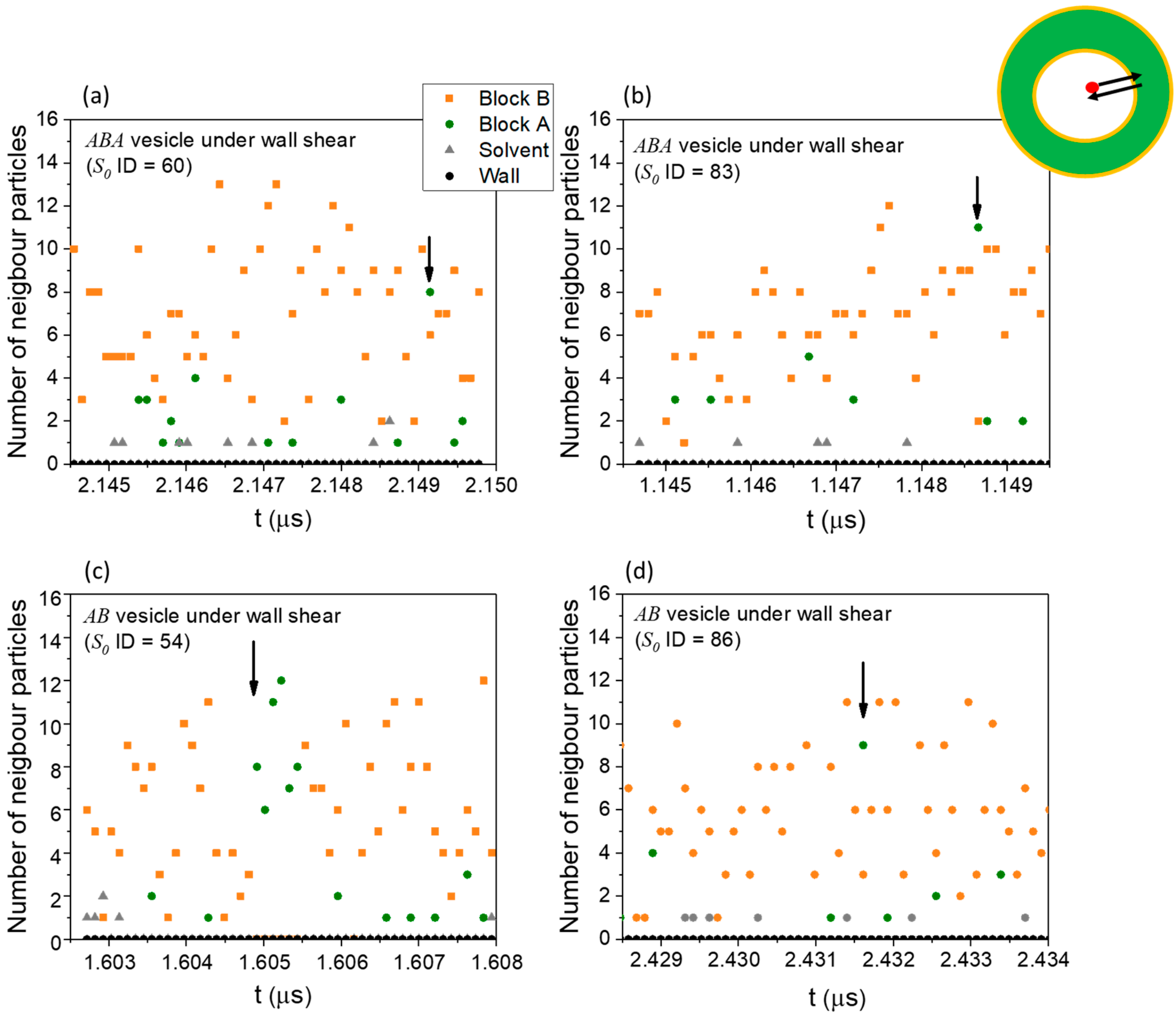

Figure S10. The numbers of *A*-, *B*- and *S*- neighbour particles vs. time of representative S_0-beads which enter the membrane briefly and then returns to cavity for (a) and (b) *ABA* and (c) and (d) *AB* vesicles under wall shear. The arrow highlights the instant when the tracked S_0-beads briefly encountered *A*-beads in the inner leaflets of the membranes.

Reference

1. Li, Z.B.; Hillmyer, M.A.; Lodge, T.P. Morphologies of multicompartment micelles formed by abc miktoarm star terpolymers. *Langmuir* **2006**, *22*, 9409-9417.
